# The complete mitochondrial genome of Indonesian snakehead, *Channa micropeltes* (Channiformes, Channidae)

**DOI:** 10.1080/23802359.2016.1199001

**Published:** 2016-08-21

**Authors:** Shoujia Jiang, Kai Zhang, Zhiqiang Ruan, Junmin Xu, Xinxin You, Qiong Shi

**Affiliations:** a Shenzhen Key Lab of Marine Genomics, Guangdong Provincial Key Lab of Molecular Breeding in Marine Economic Animals, Shenzhen, China;; b Laboratory of Aquatic Bioinformatics, BGI-Zhenjiang Institute of Hydrobiology, Zhenjiang, China

**Keywords:** *Channa micropeltes*, mitochondrial genome, phylogenetic tree

## Abstract

Here we have characterized the complete mitochondrial genome of Indonesian snakehead, *Channa micropeltes*, and described its organization in this paper. The complete mitochondrial genome, 16,567 bp, is composed of 32.54% A, 14.05% G, 25.82% T and 27.59% C. It includes 13 protein-coding genes, 22 tRNA genes, 2 rRNA genes and a 921-bp D-loop control region. Phylogenetic analysis with five more fish species demonstrated that the Indonesian snakehead is most closely related to the Great snakehead (*Channa marulius*). Our mitochondrial genomic data will also be valuable for the study on the mitochondrial evolution of fishes.

The Indonesian snakehead, *Channa micropeltes*, belonging to Channidae in Channiformes has been distributed in the fresh waters of Southeast Asia, such as Meikong River, Menam River, Malaysia peninsula, Sumatara Island and so on. It is an important food fish in the tropical Asia (Benziger et al. 2011). In this study, for the first time we reported its whole mitochondrial genome sequence (Genbank accession number KX129904).

The fish sample was collected from Sanya, China. The snakehead samples were kept at 95% ethanol before sequencing (Ruan et al. 2016). The specimen was stored at China National Genebank (Accession no. GZ2014122011). Initially, the snakehead was identified based on both the morphologic features and the COX1 sequence. Total DNA was extracted from the fins using traditional phenol–chloroform extraction method (Taggart et al. 1992). The whole genome was sequenced on the Illumina HiSeq4000 platform (San Diego, CA) at BGI-Shenzhen, China. *De novo* assemblies were generated by taking advantage of SOAP denovo-Trans (-K 71) (Tang et al. 2015). Finally, the complete mitochondrial genome is 16,567 bp in length, with the base composition of 32.54% A, 14.05% G, 25.82%T and 27.59% C. The G + C content is 41.64% and the A + T ratio is 58.36%. Further annotation by DOGMA (Wyman et al. 2004) proved that the mitochondrial genome consists of 13 protein coding genes, 22 tRNA genes and 2 rRNA genes (s-rRNA & l-rRNA) and a 921-bp D-loop control region. The D-loop is located between tRNA-Phe and tRNA-Pro. Among the mitochondrial protein-coding genes, the ND5 was the longest (1836 bp), while the ATP8 was the shortest (165 bp).

A phylogenetic tree was constructed by the neighbour-joining and maximum-likelihood methods using MEGA 6.0 (Tempe, AZ; Tamura et al., 2013) with the same topology (Figure 1). Complete mitochondrial genome sequences of five more fish species were downloaded from the NCBI. The phylogenetic analysis indicated that the Indonesian snakehead is most closely related to the Great snakehead (*Channa marulius*), which is consistent with the morphological taxonomy. We expect that our data will promote further study on the taxonomic resolution of snakeheads.

**Figure 1.**
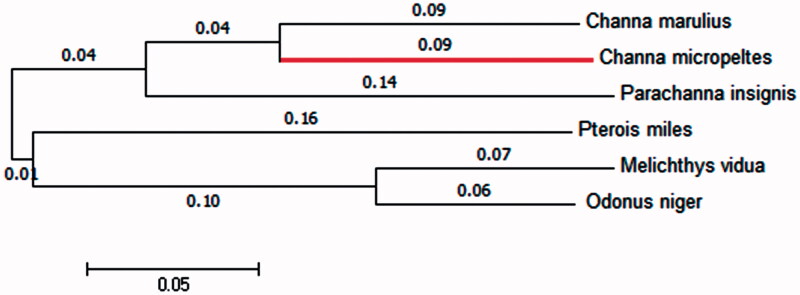
The neighbour-joining phylogenetic tree constructed on basis of the mitochondrial genome sequences of *C. micropeltes* (the red colouration) and five other fishes. Genbank accession numbers: *C. marulius*, NC_022713.1; *Parachanna insignis*, NC_022480.1; *Pterois miles*, NC_007092.3; *Melichthys_vidua*, NC_011937.1; *Odonus_niger*: NC_011938.1.
